# 

**Published:** 1982

**Authors:** 


JANUARY/APRIL 1982

VOLUME 97 (i/ii)

NUMBER 361/362

Published Quarterly

Annual Subscription £8 Post Free

### BRISTOL MEDICO-CHIRURGICAL JOURNAL

Established 1883

### BRISTOL MEDICO-CHIRURGICAL JOURNAL

Incorporating the Medical Journal of the South West

### Editorial Committee

EDITOR
*M. G. Wilson, ChM, FRCS*
*Litfield House, Clifton, Bristol, BS8 3LS*

ASSISTANT EDITOR
*Dr. G. R. Tribe*
*Southmead Hospital, Bristol, BS10 5NB*

MEMBERS
*Dr. M. B. Lennard*
*Dr. D. W. Wright*
*The Hon. Treasurer of the Society*
*The Hon. Secretary of the Society*

SECRETARY
*Mr. B. P. Jones, The Library, Medical School, University Walk, Bristol, BS8 1TD*

### Bristol Medico-Chirurgical Society

PRESIDENT 1981/82
*Dr. R. P. Warin*

PRESIDENT ELECT
*Mr. H. K. Bourns*

HONORARY SECRETARY
*Dr. H. M. Norman*
*Hillside, Vicarage Lane, Barrow Gurney, Bristol*

HONORARY TREASURER
*Dr. J. Penry*
*Southmead Hospital, Bristol, BS10 5NB*

The Society was founded in 1874. In 1883 publication of the Journal began and has continued quarterly ever since then. During the years 1949 to 1962 it appeared under the title of 'The Medical Journal of the South West'.

### Notice to Contributors

*The Journal is especially concerned to provide a place for the recording of medical thought, interests, and practice in and around Bristol. It therefore welcomes articles that, as well as being of immediate interest to members, will document the local and contemporary medical scene for our successors.*

*Original articles are invited provided that they have not been published elsewhere. Illustrations should be supplied ready for reproduction. Line drawings should be in Indian ink on firm white paper or board. The author will be informed if it is found necessary to ask him to pay for the printing of photographs, etc.*

*The following contributions will be especially welcome: notes of forthcoming events, meetings held, degrees conferred, staff appointments, honours and public appointments and other local news.*

*Advice on preparation can be obtained from the Editor.*

Printed by the University of Bristol Printing Unit

### University of Bristol Faculty of Medicine

**THE DEGREE OF DOCTOR OF MEDICINE**
**October 1981**
**Dissertations Approved**

Philip Hugh POWELL

**THE DEGREE OF MASTER OF SCIENCE IN MEDICAL SCIENCES**
**October 1981**
**Dissertation Approved**

El-Mahdy Ramadan EL-BOUESHY

**THE DEGREE OF DOCTOR OF PHILOSOPHY**
**October 1981**
**Dissertation Approved**

David Henney CROOK

**FINAL EXAMINATION FOR THE DEGREES OF M.B., Ch.B.**
**December 1981**

**PASS**
Jennifer Gillian EALAND
Mark Lloyd EVERARD
Gavin FOTHERGILL
Andrew Jeremy HARRISON
Jonathan Hugh HIGHAM
Leela Anne KALRA
Margaret Scott Douglas KERR
David Bardwell MUMFORD
Martin Graham PATTRICK

**THE DEGREE OF DOCTOR OF MEDICINE**
**January 1982**
**Dissertations Approved**

Elizabeth Mary NICHOLSON
Jerzy Marian SIKORSKI

**THE DEGREE OF DOCTOR OF PHILOSOPHY**
**January 1982**
**Dissertations Approved**

Samuel William CADDEN
James Kerr KIRKWOOD
John Anthony O'CONNOR
Judith Louise THOMPSON

**THE DEGREE OF DOCTOR OF MEDICINE**
**March 1982**
**Dissertation Approved**

Peter Campbell CLIFFORD

**THE DEGREE OF DOCTOR OF PHILOSOPHY**
**March 1982**
**Dissertations Approved**

Claire Margaret COESHOTT
Rachel ROE

**THE DEGREE OF MASTER OF SCIENCE IN MEDICAL SCIENCES**
**March 1982**

Myint KYU

